# Supplementary material for: Dietary lipid content modifies wah-1/AIFM1-associated phenotypes via LRK-1 and DRP-1 expression in C. elegans
Source: Nat Commun. 2025 Dec 1;16:10817. doi: 10.1038/s41467-025-66900-8 (PMC12669733; doi:10.1038/s41467-025-66900-8)
Supplement: Supplementary file 1 — Supplementary information [file 41467_2025_66900_MOESM1_ESM.pdf]

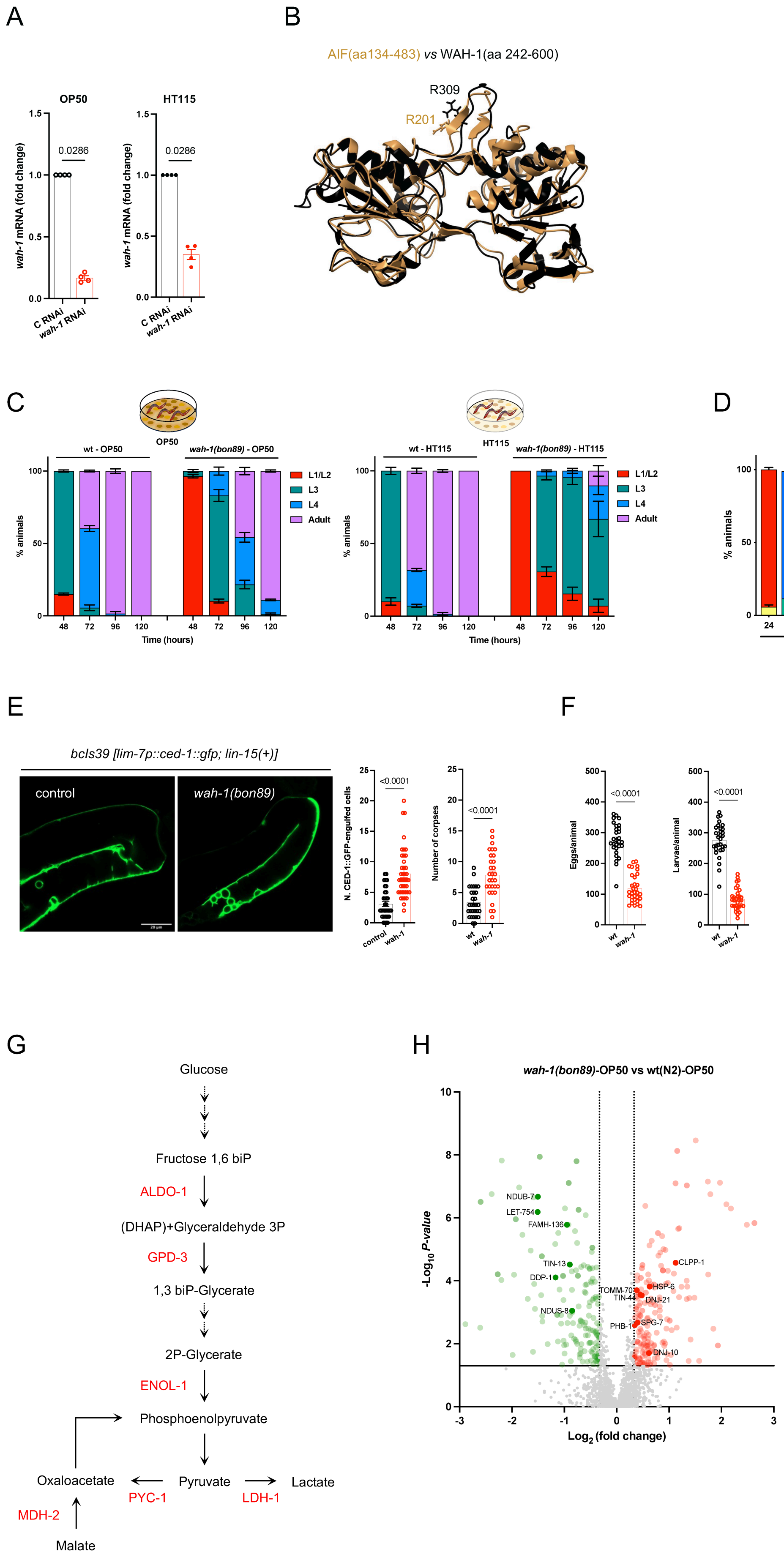

A
B
AIF(aa134-483) vs WAH-1(aa 242-600)
R309
R201
OP50
HT115
wah-1 mRNA (fold change)
0.0286
C RNAi
wah-1 RNAi
C
wt - OP50
wah-1(bon89) - OP50
wt - HT115
wah-1(bon89) - HT115
L1/L2
L3
L4
Adult
% animals
Time (hours)
D
T= 25 ºC
L1
L2
adult
wt
wah-1(bon89)
E
bcIs39 [lim-7p::ced-1::gfp; lin-15(+)]
control
wah-1(bon89)
N. CED-1::GFP-engulfed cells
Number of corpses
<0.0001
F
Eggs/animal
Larvae/animal
G
Glucose
Fructose 1,6 biP
ALDO-1
(DHAP)+Glyceraldehyde 3P
GPD-3
1,3 biP-Glycerate
2P-Glycerate
ENOL-1
Phosphoenolpyruvate
Oxaloacetate
Pyruvate
Lactate
PYC-1
LDH-1
MDH-2
Malate
H
wah-1(bon89)-OP50 vs wt(N2)-OP50
-Log10 P-value
Log2 (fold change)
NDUB-7
LET-754
FAMH-136
TIN-13
DDP-1
NDUS-8
CLPP-1
HSP-6
TOMM-70
TIN-44
DNJ-21
SPG-7
PHB-1
DNJ-10

Supplementary figure S1 (relative to Figure 1). (A) Real-time PCR analysis of *wah-1* mRNA expression in animals grown on OP50 and HT115 bacteria (n=4, Mean +/- SEM, two-tailed t-test, Mann-Whitney test). (B) 3D protein structures were predicted by AlphaFold2. Alignment of AIF and WAH-1 predicts structurally conserved catalytic domains. (C-D) Developmental assay at (C) 20°C (OP50 and HT115) and (D) 25°C (on OP50) of *wah-1(bon89)* nematodes compared to wt. On the x-axis, the time (in hours) after hatching is reported. (E) Representative confocal images of control and *wah-1(bon89)* germline expressing the *bcIs39* transgene. On the right, plots report the number of CED-1::GFP-positive engulfed cells and the number of germ-cell corpses (n=35-40 animals/conditions from 3 biological replicates, Mean +/- SEM, two-tailed t-test, Mann-Whitney test). (F) Egg laying (left) and developmental (right) assays of wt and *wah-1(bon89)* nematodes (n= 29-32 animals/condition from 3 biological replicates, Mean +/- SEM, two-tailed t-test, Mann-Whitney test). (G) Schematic representation of glycolysis and associated metabolic pathways. In red capital letters, the upregulated enzymes that have been detected in the proteomics of *wah-1(bon89)* mutants compared to wild type animals. (H) Volcano plots relative to the proteomics of *wah-1(bon89)* mutants compared to wt (N2) animals grown on OP50 bacteria. Highlight proteins are involved in mitochondrial quality control and protein import. Among the downregulated proteins, several substrates of AIF/CHCHD4 complex have been detected, including LET-754/AK2, FAMH-136, $(CX_3C)_2$ motif-containing translocases (TIN-13/TIMM13 and DDP-1/TIMM8A), and $(CX_9C)_2$ motif-containing Complex I subunits (NDUB-7/NDUFB7 and NDUFS-8/NDUFS8). Statistical analysis was performed using two-tailed t-test (thresholds: *p* value- ≤ 0.05 and FC- ± 1.25).

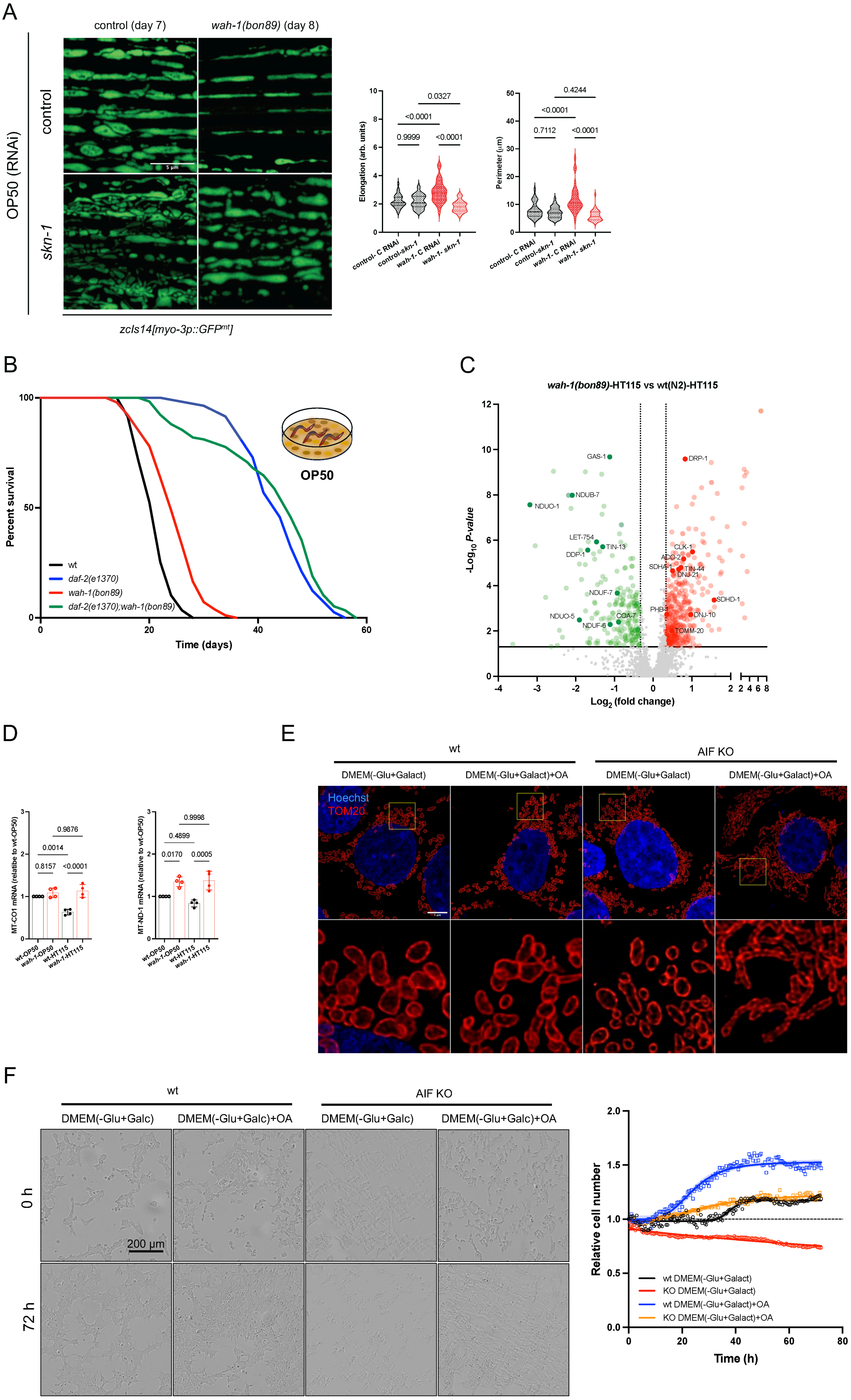
A
control (day 7)
wah-1(bon89) (day 8)
OP50 (RNAi)
control
skn-1
5 µm
zcIs14[myo-3p::GFPmt]
Elongation (arb. units)
Perimeter (µm)
control- C RNAi
control-skn-1
wah-1- C RNAi
wah-1- skn-1
0.9999
<0.0001
0.0327
0.7112
0.4244
B
Percent survival
Time (days)
wt
daf-2(e1370)
wah-1(bon89)
daf-2(e1370);wah-1(bon89)
OP50
C
wah-1(bon89)-HT115 vs wt(N2)-HT115
-Log10 P-value
Log2 (fold change)
GAS-1
NDUB-7
NDUO-1
LET-754
TIN-13
DDP-1
NDUF-7
NDUO-5
NDUF-6
COA-7
DRP-1
CLK-1
ACO-2
SDHA-1
TIN-44
DNJ-21
SDHD-1
PHB-1
DNJ-10
TOMM-20
D
MT-CO1 mRNA (relatibe to wt-OP50)
MT-ND-1 mRNA (relative to wt-OP50)
0.8157
0.0014
0.9876
0.0170
0.4899
0.9998
0.0005
wt-OP50
wah-1-OP50
wt-HT115
wah-1-HT115
E
wt
AIF KO
DMEM(-Glu+Galact)
DMEM(-Glu+Galact)+OA
Hoechst
TOM20
F
wt
AIF KO
DMEM(-Glu+Galc)
DMEM(-Glu+Galc)+OA
0 h
72 h
200 µm
Relative cell number
Time (h)
wt DMEM(-Glu+Galact)
KO DMEM(-Glu+Galact)
wt DMEM(-Glu+Galact)+OA
KO DMEM(-Glu+Galact)+OA

Supplementary figure S2 (relative to Figure 2). (A) Representative confocal images of mitochondrial networks in wt and *wah-1(bon89)* animals grown on control and *skn-1* RNAi (OP50). Analysis of mitochondrial elongation and perimeter is shown on the right (n=32-37 animals/condition from 3 biological replicates; one-way ANOVA, Šídák's multiple comparisons). (B) Representative survival curves of wt (N2), *daf-2(e1370)*, *wah-1(bon89)* and *daf-2(e1370);wah-1(bon89)* mutants on OP50 bacteria. (C) Volcano plot of dysregulated proteins in *wah-1(bon89)* compared to wt nematodes grown on HT115 (two-tailed t-test, thresholds are: p value- ≤ 0.05 and FC- ±1.25). (D) RT-PCR of MT-CO1 and MT-ND1. mRNA expression levels are relative to wt animals grown on OP50 (n=4 biological replicates, Mean +/- SEM, one-way ANOVA; Šídák's multiple comparisons). (E) Representative confocal images of wt and AIF KO HEK293T cells treated with glucose-free DMEM supplemented with galactose and glutamine, with or without oleic acid. Mitochondria were stained with TOM20 (red), and nuclei were stained with Hoechst (blue) (scale bar= 5 µm). Insets (yellow square) provide regions of interest at higher magnification (lower row of representative images). (F) Representative brightfield images of wt and AIF KO HEK293T cells treated with glucose-free DMEM supplemented with galactose and glutamine, with or without oleic acid at 0h and 72h. On the right, the plot displays cell growth/viability curves over time.

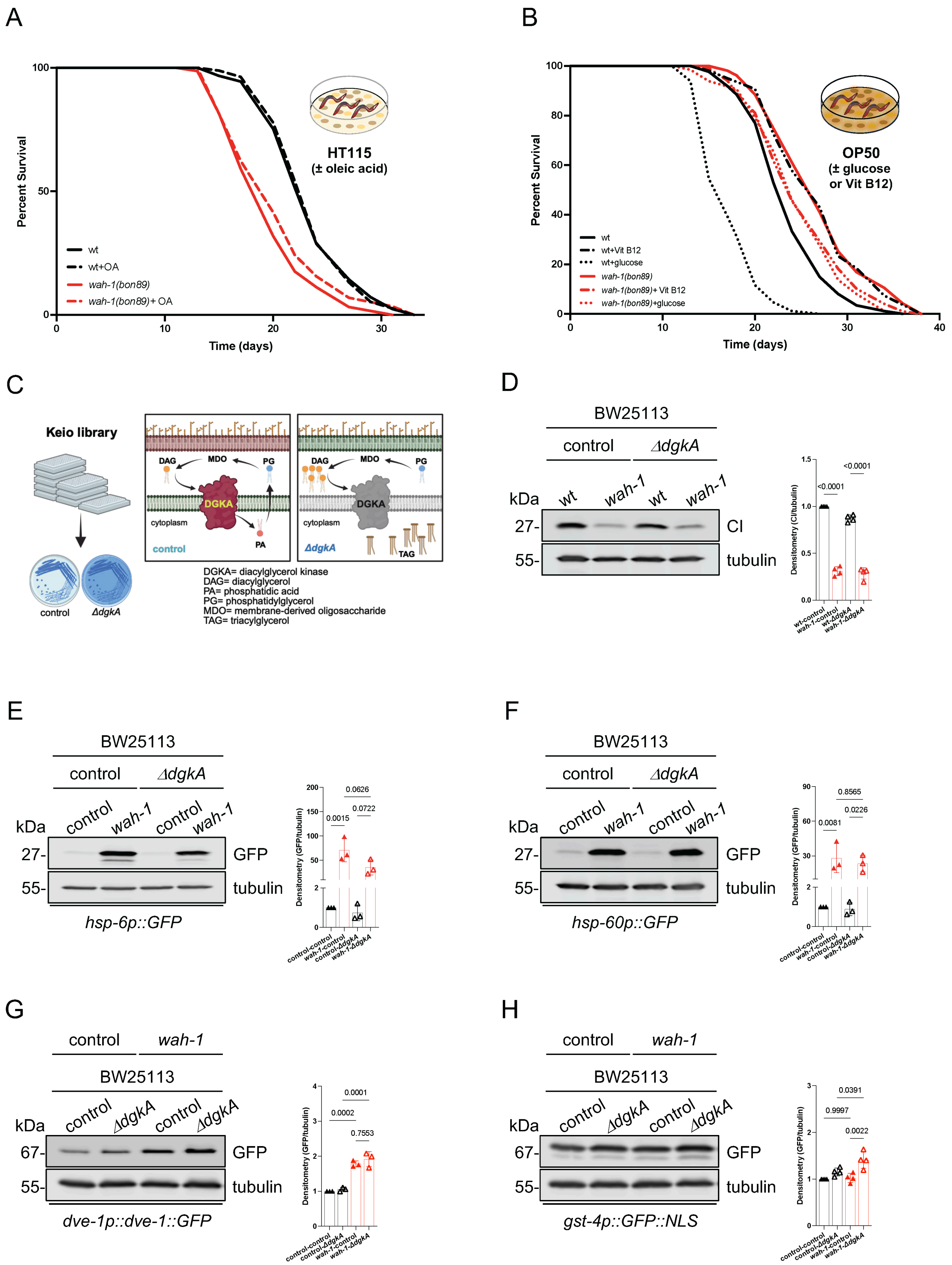
A
Percent Survival
Time (days)
HT115
(± oleic acid)
wt
wt+OA
wah-1(bon89)
wah-1(bon89)+ OA
B
Percent Survival
Time (days)
OP50
(± glucose
or Vit B12)
wt
wt+Vit B12
wt+glucose
wah-1(bon89)
wah-1(bon89)+ Vit B12
wah-1(bon89)+glucose
C
Keio library
control
ΔdgkA
DAG
MDO
PG
DGKA
cytoplasm
PA
TAG
DGKA= diacylglycerol kinase
DAG= diacylglycerol
PA= phosphatidic acid
PG= phosphatidylglycerol
MDO= membrane-derived oligosaccharide
TAG= triacylglycerol
D
BW25113
control
ΔdgkA
kDa
wt
wah-1
27-
55-
CI
tubulin
Densitometry (CI/tubulin)
<0.0001
<0.0001
wt-control
wah-1-control
wt-ΔdgkA
wah-1-ΔdgkA
E
BW25113
control
ΔdgkA
kDa
control
wah-1
27-
55-
GFP
tubulin
hsp-6p::GFP
Densitometry (GFP/tubulin)
0.0015
0.0626
0.0722
control-control
wah-1-control
control-ΔdgkA
wah-1-ΔdgkA
F
BW25113
control
ΔdgkA
kDa
control
wah-1
27-
55-
GFP
tubulin
hsp-60p::GFP
Densitometry (GFP/tubulin)
0.0081
0.8565
0.0226
control-control
wah-1-control
control-ΔdgkA
wah-1-ΔdgkA
G
control
wah-1
BW25113
kDa
control
ΔdgkA
67-
55-
GFP
tubulin
dve-1p::dve-1::GFP
Densitometry (GFP/tubulin)
0.0002
0.0001
0.7553
control-control
control-ΔdgkA
wah-1-control
wah-1-ΔdgkA
H
control
wah-1
BW25113
kDa
control
ΔdgkA
67-
55-
GFP
tubulin
gst-4p::GFP::NLS
Densitometry (GFP/tubulin)
0.9997
0.0391
0.0022
control-control
control-ΔdgkA
wah-1-control
wah-1-ΔdgkA

Supplementary figure S3 (relative to Figure 3). (A) Representative survival curves of wt (N2) and *wah-1(bon89)* animals grown on HT115 bacteria supplemented with oleic acid (OA). (B) Representative survival curves of wt (N2) and *wah-1(bon89)* animals grown on OP50 bacteria supplemented with vitamin B12 or glucose. (C) Schematic representation of the Keio library and the molecular function of DGKA enzyme. Created in BioRender. Bano, D. (2026) https://BioRender.com/xbjr3z5. (D) Representative immunoblots of NUO-2/NDUFS3 (CI) and tubulin (as a loading control). Samples are from wt and *wah-1(bon89)* mutant animals grown on BW25113 strains. Densitometry is reported on the right (n=4, Mean +/- SEM, one-way ANOVA, Tukey's multiple comparisons test). (E-H) Representative immunoblots of GFP and tubulin of samples from control and *wah-1* mutants grown on *E. coli* K-12 control and *ΔdgkA*. Densitometry analysis of (E) *[hsp-6p::GFP]*, (F) *[hsp-60p::GFP]*, (G) *[dve-1p::dve-1::GFP]* and (H) *[gst-4p::GFP::NLS]* are shown next to the immunoblots (n=3-4, Mean +/- SEM, one-way ANOVA, Šídák's multiple comparisons test).

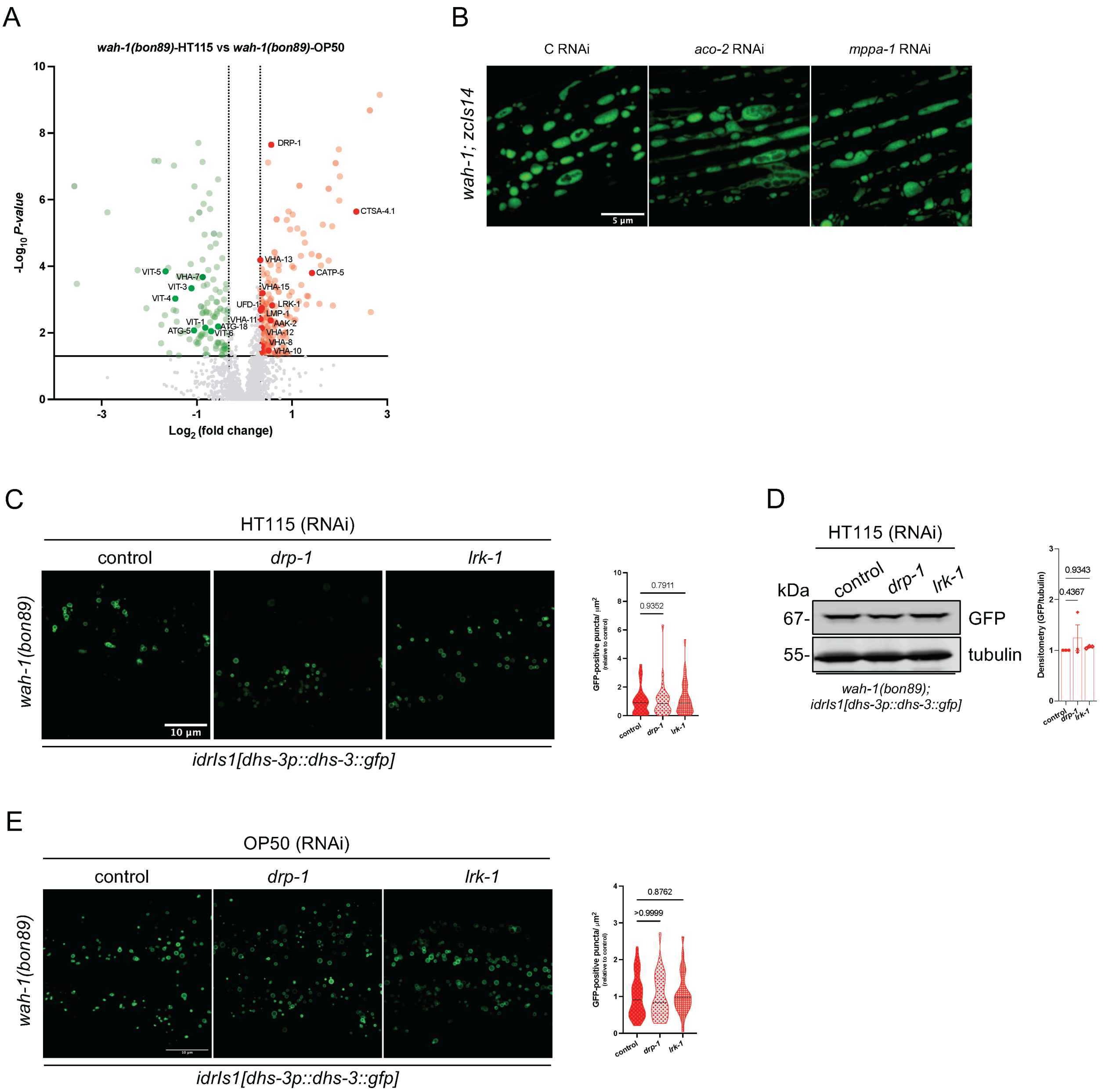
A
wah-1(bon89)-HT115 vs wah-1(bon89)-OP50
-Log10 P-value
Log2 (fold change)
DRP-1
CTSA-4.1
VHA-13
CATP-5
VHA-15
LRK-1
UFD-1
LMP-1
AAK-2
VHA-12
VHA-8
VHA-10
VHA-11
VIT-5
VHA-7
VIT-3
VIT-4
VIT-1
ATG-18
ATG-5
VIT-6
B
C RNAi
aco-2 RNAi
mppa-1 RNAi
wah-1; zcIs14
5 µm
C
HT115 (RNAi)
control
drp-1
lrk-1
wah-1(bon89)
10 µm
idrIs1[dhs-3p::dhs-3::gfp]
GFP-positive puncta/ µm2 (relative to control)
0.9352
0.7911
D
HT115 (RNAi)
kDa
control
drp-1
lrk-1
67-
55-
GFP
tubulin
wah-1(bon89); idrIs1[dhs-3p::dhs-3::gfp]
Densitometry (GFP/tubulin)
0.4367
0.9343
E
OP50 (RNAi)
control
drp-1
lrk-1
wah-1(bon89)
idrIs1[dhs-3p::dhs-3::gfp]
GFP-positive puncta/ µm2 (relative to control)
>0.9999
0.8762

Supplementary figure S4 (relative to Figure 4). (A) Differentially regulated proteins in *wah-1* mutants grown either in HT115 or OP50. Upregulated proteins include lysosomal proteins (CTSA-4.1, CATP-5, LMP-1). Among the dysregulated proteins, there are autophagy-related proteins (ATG-18 and ATG-5) and egg yolk precursors (vitellogenin VIT-3, VIT-4, VIT-5, VIT-6). Moreover, many vacuolar H+ ATPase subunits (VHA-7, VHA-8, VHA-10, VHA-11, VHA-12, VHA-15, VHA-13) part of the lysosomal surveillance response were detected. (two-tailed t-test, thresholds are: *p* value- ≤ 0.05 and FC- ± 1.25). (B) Representative confocal images of mitochondria in *wah-1* mutants expressing *zcIs14[myo-3p::GFP(mt)]* and grown on control, *aco-2* and *mppa-1* RNAi (HT115 bacteria). (C) Confocal images of DHS-3::GFP of *wah-1* mutants expressing *idrIs1[dhs-3p::dhs-3::gfp]* grown on control, *drp-1* and *lrk-1* RNAi (HT115 bacteria). Quantification of GFP signal is provided on the right (n=30 animals/condition from 3 biological replicates, one-way ANOVA, Dunnett's multiple comparisons test). (D) Representative immunoblots of samples from *wah-1* mutants expressing *idrIs1[dhs-3p::dhs-3::gfp]* grown on HT115 expressing control, *drp-1* and *lrk-1* RNAi. Densitometry is reported on the right (n=3, one-way ANOVA, Dunnett's multiple comparisons test, ns= not significant). (E) Representative confocal images of DHS-3::GFP of *wah-1(bon89)* animals expressing *idrIs1[dhs-3p::dhs-3::gfp]* grown on OP50 bacteria expressing control, *drp-1* and *lrk-1* RNAi. On the right, quantification of GFP signal is shown (n= 30 animals/condition from 3 biological replicates, one-way ANOVA, Dunnett's multiple comparisons test).
