# Supplementary material for: Dietary lipid content modifies wah-1/AIFM1-associated phenotypes via LRK-1 and DRP-1 expression in C. elegans
Source: Nat Commun. 2025 Dec 1;16:10817. doi: 10.1038/s41467-025-66900-8 (PMC12669733; doi:10.1038/s41467-025-66900-8)
Supplement: Supplementary file 2 — Description of Additional Supplementary File [file 41467_2025_66900_MOESM2_ESM.pdf]

Description of Additional Supplementary Files

Supplementary data 1: Metabolomic analysis of E. coli-type strain OP50, K-12 strain HT115(DE3), K-12 control (BW25113 strain) and DdgkA bacteria. Experiments are performed with four biological replicates.

Supplementary data 2: Proteomics analysis of wah-1(bon89) compared to wt nematodes in OP50 bacteria. (n=4, two-tailed t-test, p-values were corrected using the Benjamini-Hochberg (BH) false discovery rate).

Supplementary data 3: Proteomics analysis of wt and wah-1(bon89) nematodes grown on HT115 bacteria. (n=4, two-tailed t-test, p-values were corrected using the Benjamini-Hochberg (BH) false discovery rate).

Supplementary data 4: Proteome change in wah-1(bon89) grown on HT115 compared to wah-1(bon89) grown on OP50. (n=4, two-tailed t-test, p-values were corrected using the Benjamini-Hochberg (BH) false discovery rate).

Supplementary data 5: Lipidomic analysis of wah-1(bon89) grown on HT115 in comparison to wah-1(bon89) grown on OP50. (n=4, two-tailed t-test, the resulting p-values were further adjusted using the Benjamini-Hochberg (BH)).

Supplementary data 6: Lipidomic analysis of wah-1 mutants grown on E. coli K-12 DdgkA or control (BW25113). (n=4, two-tailed t-test, the resulting p-values were further adjusted using the Benjamini-Hochberg (BH)).

Supplementary Data 7. A summary and detailed tables for each lifespan experiment are reported with statistical analyses. The means of the median survival (mean) +/- standard error of the mean (SEM) are calculated based on the biological replicates for the indicated treatments (bacteria strains, RNAi clones) and genotypes.

Supplementary Data 8. Individual lifespan of HT115 RNAi screening against upregulated metabolic enzymes and mitochondrial-associated proteins in wt and *wah-1(bon89)*.
