# Supplementary material for: Dietary lipid content modifies wah-1/AIFM1-associated phenotypes via LRK-1 and DRP-1 expression in C. elegans
Source: Nat Commun. 2025 Dec 1;16:10817. doi: 10.1038/s41467-025-66900-8 (PMC12669733; doi:10.1038/s41467-025-66900-8)
Supplement: Supplementary file 9 — Supplementary data 7 [file 41467_2025_66900_MOESM9_ESM.zip › AIF-WAH-1_Supplementary data 7.docx]

**Supplementary data 7.**

**Lifespan data summary (statistics based on biological replicates).** Individual tables report statistical analyses of lifespan data. The means of the median survival (mean)± standard error of the mean (SEM) are calculated based on the biological replicates for the indicated treatments (bacteria strains, RNAi clones) and genotypes. Individual experiments are reported below in “Summary tables for individual assays”.

| **OP50(xu363)** | | |
| --- | --- | --- |
| **Genotypes and treatments** | **Mean±SEM** | **Replicates** |
| wt (N2)- C RNAi | 24.33 ± 0.25 | 3 |
| wt (N2)- *wah-1* RNAi | 26.67 ± 0.25 | 3 |

| **HT115** | | |
| --- | --- | --- |
| **Genotypes and treatments** | **Mean±SEM** | **Replicates** |
| wt (N2)- C RNAi | 24.00 ± 0.80 | 4 |
| wt (N2)- *wah-1* RNAi | 22.33 ± 0.50 | 3 |
| *wah-1(bon89)-* C RNAi | 20.50 ± 0.22 | 4 |

| **OP50** | | |
| --- | --- | --- |
| **Genotypes and treatments** | **Mean±SEM** | **Replicates** |
| wt (N2) | 22.60 ± 0.21 | 5 |
| *wah-1(bon89)* | 24.75 ± 0.20 | 4 |
| *daf-2(e1370)* | 45.33 ± 0.25 | 3 |
| *daf-2(e1370);wah-1(bon89)* | 46.00 ± 0.75 | 3 |

| **OP50** | | |
| --- | --- | --- |
| **Genotypes and treatments** | **Mean±SEM** | **Replicates** |
| wt (N2) | 24.00 ± 0.00 | 3 |
| *atfs-1(tm4525)* | 24.67 ± 0.25 | 3 |
| *wah-1(bon89)* | 27.33 ± 0.25 | 3 |
| *wah-1(bon89);atfs-1(tm4525)* | 26.67 ± 0.25 | 3 |

| **OP50(xu363)** | | |
| --- | --- | --- |
| **Genotypes and treatments** | **Mean±SEM** | **Replicates** |
| wt (N2)- C RNAi | 24.33 ± 0.50 | 3 |
| wt (N2)- *skn-1* RNAi | 25.00 ± 0.00 | 3 |
| *wah-1(bon89)-* C RNAi | 28.00 ± 0.00 | 3 |
| *wah-1(bon89)- skn-1* RNAi | 26.00 ± 0.00 | 3 |

| **HT115** | | |
| --- | --- | --- |
| **Genotypes and treatments** | **Mean±SEM** | **Replicates** |
| wt (N2) | 24.67 ± 0.25 | 3 |
| wt (N2)+ oleic acid (OA) | 24.67 ± 0.25 | 3 |
| *wah-1(bon89)* | 19.33 ± 0.50 | 3 |
| *wah-1(bon89)*+ oleic acid (OA) | 21.00 ± 0.75 | 3 |

| **OP50** | | |
| --- | --- | --- |
| **Genotypes and treatments** | **Mean±SEM** | **Replicates** |
| wt (N2) | 24.00 ± 0.00 | 3 |
| wt (N2)+ Vitamin B12 | 27.00 ± 0.00 | 3 |
| wt (N2)+ glucose | 17.00 ± 0.00 | 3 |
| *wah-1(bon89)* | 27.00 ± 0.00 | 3 |
| *wah-1(bon89)*+ Vitamin B12 | 25.00 ± 0.75 | 3 |
| *wah-1(bon89)*+ glucose | 24.00 ± 0.00 | 3 |

| **BW25113** | | |
| --- | --- | --- |
| **Genotypes and treatments** | **Mean±SEM** | **Replicates** |
| wt (N2)- control | 23.00 ± 0.41 | 3 |
| wt (N2)- *ΔdgkA* | 23.00 ± 0.41 | 3 |
| *wah-1(bon89)*- control | 17.33 ± 0.25 | 3 |
| *wah-1(bon89)*- *ΔdgkA* | 25.33 ± 0.63 | 3 |

| **HT115** | | |
| --- | --- | --- |
| **Genotypes and treatments** | **Mean±SEM** | **Replicates** |
| wt (N2)- C RNAi | 24.33 ± 0.25 | 3 |
| wt (N2)- *lrk-1* RNAi | 24.33 ± 0.25 | 3 |
| wt (N2)- *drp-1* RNAi | 23.67 ± 0.25 | 3 |
| *wah-1(bon89)-* C RNAi | 19.67 ± 0.25 | 3 |
| *wah-1(bon89)*- *lrk-1* RNAi | 23.67 ± 0.25 | 3 |
| *wah-1(bon89)*- *drp-1* RNAi | 23.67 ± 0.25 | 3 |

| **HT115** | | |
| --- | --- | --- |
| **Genotypes and treatments** | **Mean±SEM** | **Replicates** |
| wt (N2)- C RNAi | 24.33 ± 0.25 | 3 |
| wt (N2)- *pink-1* RNAi | 23.67 ± 0.25 | 3 |
| *wah-1(bon89)-* C RNAi | 20.00 ± 0.00 | 3 |
| *wah-1(bon89)-* *pink-1* RNAi | 22.33 ± 0.25 | 3 |

| **HT115** | | |
| --- | --- | --- |
| **Genotypes and treatments** | **Mean±SEM** | **Replicates** |
| wt (N2)- C RNAi | 24.00 ± 0.00 | 3 |
| wt (N2)- *lrk-1* RNAi | 24.00 ± 0.00 | 3 |
| *drp-1(tm1108)*- C RNAi | 21.67 ± 0.25 | 3 |
| *drp-1(tm1108)-* *lrk-1* RNAi | 22.00 ± 0.82 | 3 |
| *wah-1(bon89)*- C RNAi | 19.00 ± 0.00 | 3 |
| *wah-1(bon89)*- *lrk-1* RNAi | 23.67 ± 0.25 | 3 |
| *wah-1(bon89);drp-1(tm1108)*- C RNAi | 24.33 ± 0.25 | 3 |
| *wah-1(bon89);drp-1(tm1108)*- *lrk-1* RNAi | 24.33 ± 0.25 | 3 |

| **OP50(xu363)** | | |
| --- | --- | --- |
| **Genotypes and treatments** | **Mean±SEM** | **Replicates** |
| wt (N2)- C RNAi | 24.00 ± 0.00 | 3 |
| wt (N2)- *wah-1* RNAi | 26.67 ± 0.50 | 3 |
| *DRP-1 O/E-* C RNAi | 24.00 ± 0.75 | 3 |
| *DRP-1 O/E-* *wah-1* RNAi | 21.33 ± 0.25 | 3 |

| **HT115** | | |
| --- | --- | --- |
| **Genotypes and treatments** | **Mean±SEM** | **Replicates** |
| wt (N2)- C RNAi | 24.25 ± 0.02 | 4 |
| wt (N2)- *drp-1* RNAi | 23.25 ± 0.37 | 4 |
| *lrk-1(bon150)*- C RNAi | 24.25 ± 0.20 | 4 |
| *lrk-1(bon150)- drp-1* RNAi | 24.25 ± 0.20 | 4 |
| *lrk-1(bon151)*- C RNAi | 24.25 ± 0.20 | 4 |
| *lrk-1(bon151)-* *drp-1* RNAi | 24.25 ± 0.20 | 4 |
| *lrk-1(bon150);wah-1(bon89)*- C RNAi | 24.25 ± 0.20 | 4 |
| *lrk-1(bon150);wah-1(bon89)-* *drp-1* RNAi | 24.75 ± 0.37 | 4 |
| *lrk-1(bon151);wah-1(bon89)*- C RNAi | 25.25 ± 0.37 | 4 |
| *lrk-1(bon151);wah-1(bon89)-* *drp-1* RNAi | 24.75 ± 0.37 | 4 |

**Summary tables for individual assays.** Tables report genotypes, number of animals (dead and censored subjects), median survival (days) and max (days) lifespan for each individual experiment (Exp). To calculate *p* values, Log-rank (Mantel-Cox) test was used. Below each table, the comparison and relative *p* value are reported.

| **OP50 (xu363)** | | | | | |
| --- | --- | --- | --- | --- | --- |
| **Exp** | **Genotypes and treatments** | **Dead (Censored)** | **Median** | **Max** | ***p* value** |
| 1 | wt (N2)- C RNAi | 106(21) | 25 | 33 | - |
|  | wt (N2)- *wah-1* RNAi | 94(29) | 27 | 37 | 0.0001^a^ |
| 2 | wt (N2)- C RNAi | 105(21) | 24 | 33 | - |
|  | wt (N2)- *wah-1* RNAi | 82(32) | 27 | 37 | <0.0001^a^ |
| 3 | wt (N2)- C RNAi | 119(15) | 24 | 35 | - |
|  | wt (N2)- *wah-1* RNAi | 90(43) | 26 | 37 | <0.0001^a^ |

^a^*p* value compared to wt (N2)- C RNAi

| **HT115** | | | | | |
| --- | --- | --- | --- | --- | --- |
| **Exp** | **Genotypes and treatments** | **Dead (Censored)** | **Median** | **Max** | ***p* value** |
| 4 | wt (N2)- C RNAi | 65(15) | 21 | 30 | - |
|  | *wah-1(bon89)-* C RNAi | 104(76) | 20 | 28 | 0.0392^a^ |
| 5 | wt (N2)- C RNAi | 56(20) | 25 | 33 | - |
|  | wt (N2)- *wah-1* RNAi | 94(24) | 23 | 33 | 0.0001^a^ |
|  | *wah-1(bon89)-* C RNAi | 80(43) | 21 | 34 | 0.0004^a^ |
| 6 | wt (N2)- C RNAi | 119(29) | 25 | 33 | - |
|  | wt (N2)- *wah-1* RNAi | 147(15) | 23 | 31 | 0.0055^a^ |
|  | *wah-1(bon89)-* C RNAi | 76(75) | 20 | 31 | <0.0001^a^ |
| 7 | wt (N2)- C RNAi | 81(36) | 25 | 33 | - |
|  | wt (N2)- *wah-1* RNAi | 129(47) | 21 | 31 | <0.0001^a^ |
|  | *wah-1(bon89)-* C RNAi | 70(70) | 21 | 31 | <0.0001^a^ |

^a^*p* value compared to wt (N2)- C RNAi

| **OP50** | | | | | |
| --- | --- | --- | --- | --- | --- |
| **Exp** | **Genotypes and treatments** | **Dead (Censored)** | **Median** | **Max** | ***p* value** |
| 8 | wt (N2) | 59(18) | 22 | 28 | - |
|  | *wah-1(bon89)* | 183(77) | 24 | 36 | <0.0001^a^ |
|  | *daf-2(e1370)* | 81(21) | 46 | 56 | <0.0001^a^  <0.0001^b^ |
|  | *daf-2(e1370);wah-1(bon89)* | 116(133) | 44 | 58 | <0.0001^a^  <0.0001^b^  ns^c^ |
| 9 | wt (N2) | 94(26) | 22 | 30 | - |
|  | *wah-1(bon89)* | 121(45) | 25 | 34 | <0.0001^a^ |
| 10 | wt (N2) | 77(23) | 23 | 32 | - |
|  | *wah-1(bon89)* | 126(54) | 25 | 37 | 0.0005^a^ |
| 11 | wt (N2) | 74(24) | 23 | 33 | - |
|  | *wah-1(bon89)* | 117(53) | 25 | 41 | <0.0001^a^ |
|  | *daf-2(e1370)* | 135(45) | 45 | 61 | <0.0001^a^  <0.0001^b^ |
|  | *daf-2(e1370);wah-1(bon89)* | 87(104) | 47 | 61 | <0.0001^a^  <0.0001^b^  ns^c^ |
| 12 | wt (N2) | 86(45) | 23 | 34 | - |
|  | *daf-2(e1370)* | 125(47) | 45 | 60 | <0.0001^a^ |
|  | *daf-2(e1370);wah-1(bon89)* | 70(141) | 47 | 62 | <0.0001^a^  0.0182^b^ |

^a^*p* value compared to wt (N2)

^b^*p* value compared to *wah-1(bon89)*

^c^*p* value compared to *daf-2(e1370)*

| **OP50** | | | | | |
| --- | --- | --- | --- | --- | --- |
| **Exp** | **Genotypes and treatments** | **Dead (Censored)** | **Median** | **Max** | ***p* value** |
| 13 | wt (N2) | 98(24) | 24 | 32 | - |
|  | *atfs-1(tm4525)* | 100(25) | 25 | 35 | 0.0229^a^ |
|  | *wah-1(bon89)* | 101(49) | 27 | 37 | <0.0001^a^ |
|  | *wah-1(bon89);atfs-1(tm4525)* | 114(36) | 27 | 37 | <0.0001^a^  ns^b^ |
| 14 | wt (N2) | 88(37) | 24 | 32 | - |
|  | *atfs-1(tm4525)* | 97(28) | 25 | 33 | 0.0190^a^ |
|  | *wah-1(bon89)* | 100(50) | 27 | 37 | <0.0001^a^ |
|  | *wah-1(bon89);atfs-1(tm4525)* | 113(37) | 27 | 37 | <0.0001^a^  ns^b^ |
| 15 | wt (N2) | 103(22) | 24 | 34 | - |
|  | *atfs-1(tm4525)* | 107(17) | 24 | 34 | ns^a^ |
|  | *wah-1(bon89)* | 99(51) | 28 | 36 | <0.0001^a^ |
|  | *wah-1(bon89);atfs-1(tm4525)* | 118(32) | 26 | 36 | <0.0001^a^  ns^b^ |

^a^*p* value compared to wt (N2)

^b^*p* value compared to *wah-1(bon89)*

| **OP50(xu363)** | | | | | |
| --- | --- | --- | --- | --- | --- |
| **Exp** | **Genotypes and treatments** | **Dead (Censored)** | **Median** | **Max** | ***p* value** |
| 16 | wt (N2)- C RNAi | 97(25) | 23 | 33 | - |
|  | wt (N2)- *skn-1* RNAi | 94(26) | 25 | 33 | ns^a^ |
|  | *wah-1(bon89)-* C RNAi | 91(59) | 28 | 38 | <0.0001^a^ |
|  | *wah-1(bon89)-* *skn-1* RNAi | 116(34) | 26 | 36 | 0.0074^a^  0.0002^b^ |
| 17 | wt (N2)- C RNAi | 85(30) | 25 | 35 | - |
|  | wt (N2)- *skn-1* RNAi | 91(32) | 25 | 33 | 0.0004^a^ |
|  | *wah-1(bon89)*- C RNAi | 77(73) | 28 | 36 | <0.0001^a^ |
|  | *wah-1(bon89)-* *skn-1* RNAi | 110(40) | 26 | 36 | ns^a^  <0.0001^b^ |
| 18 | wt (N2)- C RNAi | 125(15) | 25 | 33 | - |
|  | wt (N2)- *skn-1* RNAi | 113(24) | 25 | 33 | ns^a^ |
|  | *wah-1(bon89)-* C RNAi | 75(78) | 28 | 38 | <0.0001^a^ |
|  | *wah-1(bon89)- skn-1* RNAi | 78(82) | 26 | 36 | 0.0185^a^  0.0017^b^ |

^a^*p* value compared to wt (N2)- C RNAi

^b^*p* value compared to *wah-1(bon89)*- C RNAi

| **HT115** | | | | | |
| --- | --- | --- | --- | --- | --- |
| **Exp** | **Genotypes and treatments** | **Dead (Censored)** | **Median** | **Max** | ***p* value** |
| 19 | wt (N2) | 84(24) | 24 | 33 | - |
|  | wt (N2)+ oleic acid (OA) | 76(30) | 24 | 33 | ns^a^ |
|  | *wah-1(bon89)* | 63(71) | 20 | 31 | <0.0001^a^  - |
|  | *wah-1(bon89)*+ oleic acid (OA) | 58(82) | 20 | 33 | <0.0001^a^  ns^b^ |
| 20 | wt (N2) | 81(26) | 25 | 35 | - |
|  | wt (N2)+ oleic acid (OA) | 72(33) | 25 | 35 | ns^a^ |
|  | *wah-1(bon89)* | 89(51) | 18 | 29 | <0.0001^a^  - |
|  | *wah-1(bon89)*+ oleic acid (OA) | 92(45) | 20 | 31 | <0.0001^a^  ns^b^ |
| 21 | wt (N2) | 91(35) | 25 | 35 | - |
|  | wt (N2)+ oleic acid (OA) | 77(31) | 25 | 35 | ns^a^ |
|  | *wah-1(bon89)* | 97(42) | 20 | 31 | <0.0001^a^  - |
|  | *wah-1(bon89)*+ oleic acid (OA) | 89(43) | 23 | 31 | <0.0001^a^  0.0293^b^ |

^a^*p* value compared to wt (N2)

^b^*p* value compared to *wah-1(bon89)*

| **OP50** | | | | | |
| --- | --- | --- | --- | --- | --- |
| **Exp** | **Genotypes and treatments** | **Dead (Censored)** | **Median** | **Max** | ***p* value** |
| 22 | wt (N2) | 87(21) | 24 | 36 | - |
|  | wt (N2)+ Vit B12 | 84(46) | 27 | 38 | <0.0001^a^ |
|  | wt (N2)+ glucose | 87(45) | 17 | 27 | <0.0001^a^ |
|  | *wah-1(bon89)* | 97(65) | 27 | 38 | <0.0001^a^ |
|  | *wah-1(bon89)*+ Vit B12 | 99(51) | 24 | 38 | 0.0210^a^  0.0169^b^ |
|  | *wah-1(bon89)*+ glucose | 89(66) | 24 | 36 | ns^a^  0.0028^b^ |
| 23 | wt (N2) | 78(25) | 24 | 35 | - |
|  | wt (N2)+ Vit B12 | 77(23) | 27 | 37 | 0.0035^a^ |
|  | wt (N2)+ glucose | 65(34) | 17 | 27 | <0.0001^a^ |
|  | *wah-1(bon89)* | 87(52) | 27 | 37 | 0.0002^a^ |
|  | *wah-1(bon89)*+ Vit B12 | 100(44) | 27 | 35 | ns^a^  0.0164^b^ |
|  | *wah-1(bon89)*+ glucose | 86(65) | 24 | 35 | ns^a^  0.001^b^ |
| 24 | wt (N2) | 88(23) | 24 | 35 | - |
|  | wt (N2)+ Vit B12 | 71(39) | 27 | 37 | 0.0008^a^ |
|  | wt (N2)+ glucose | 65(35) | 17 | 29 | <0.0001^a^ |
|  | *wah-1(bon89)* | 93(58) | 27 | 37 | 0.0027^a^ |
|  | *wah-1(bon89)*+ Vit B12 | 88(52) | 24 | 35 | ns^a^  ns^b^ |
|  | *wah-1(bon89)*+ glucose | 89(57) | 24 | 35 | 0.039^a^  ns^b^ |

^a^*p* value compared to wt (N2)

^b^*p* value compared to *wah-1(bon89)*

| **BW25113** | | | | | |
| --- | --- | --- | --- | --- | --- |
| **Exp** | **Genotypes and treatments** | **Dead (Censored)** | **Median** | **Max** | ***p* value** |
| 25 | wt (N2)- control | 79(39) | 23 | 33 | - |
|  | wt (N2)- *ΔdgkA* | 99(20) | 23 | 35 | ns^a^ |
|  | *wah-1(bon89)*- control | 98(112) | 17 | 27 | <0.0001^a^  - |
|  | *wah-1(bon89)*- *ΔdgkA* | 123(84) | 25 | 37 | 0.0135^a^  <0.0001^b^ |
| 26 | wt (N2)- control | 66(34) | 22 | 33 | - |
|  | wt (N2)- *ΔdgkA* | 90(34) | 22 | 33 | ns^a^ |
|  | *wah-1(bon89)*- control | 87(81) | 17 | 29 | <0.0001^a^  - |
|  | *wah-1(bon89)*- *ΔdgkA* | 100(65) | 24 | 35 | 0.0044^a^  <0.0001^b^ |
| 27 | wt (N2)- control | 73(50) | 24 | 33 | - |
|  | wt (N2)- *ΔdgkA* | 65(62) | 24 | 33 | ns^a^ |
|  | *wah-1(bon89)*- control | 109(57) | 18 | 32 | <0.0001^a^  - |
|  | *wah-1(bon89)*- *ΔdgkA* | 103(60) | 27 | 36 | <0.0001^a^  <0.0001^b^ |

^a^*p* value compared to wt (N2)- control

^b^*p* value compared to *wah-1(bon89)*- control

| **HT115** | | | | | |
| --- | --- | --- | --- | --- | --- |
| **Exp** | **Genotypes and treatments** | **Dead (Censored)** | **Median** | **Max** | ***p* value** |
| 28 | wt (N2)- C RNAi | 119(29) | 25 | 36 | - |
|  | wt (N2)- *lrk-1* RNAi | 113(38) | 25 | 36 | ns^a^ |
|  | wt (N2)- *drp-1* RNAi | 101(16) | 23 | 34 | 0.0131^a^ |
|  | *wah-1(bon89)*- C RNAi | 84(114) | 19 | 29 | <0.0001^a^ |
|  | *wah-1(bon89)*- *lrk-1* RNAi | 145(60) | 23 | 36 | 0.0198^a^  <0.0001^b^ |
|  | *wah-1(bon89)*- *drp-1* RNAi | 130(49) | 23 | 34 | 0.0052^a^  <0.0001^b^ |
| 29 | wt (N2)- C RNAi | 109(34) | 24 | 34 | - |
|  | wt (N2)- *lrk-1* RNAi | 131(16) | 24 | 34 | ns^a^ |
|  | wt (N2)- *drp-1* RNAi | 121(23) | 24 | 32 | 0.0066^a^ |
|  | *wah-1(bon89)*- C RNAi | 147(68) | 20 | 30 | <0.0001^a^ |
|  | *wah-1(bon89)*- *lrk-1* RNAi | 192(25) | 24 | 34 | ns^a^  <0.0001^b^ |
|  | *wah-1(bon89)*- *drp-1* RNAi | 206(12) | 24 | 34 | ns^a^  <0.0001^b^ |
| 30 | wt (N2)- C RNAi | 126(27) | 24 | 35 | - |
|  | wt (N2)- *lrk-1* RNAi | 110(38) | 24 | 35 | ns^a^ |
|  | wt (N2)- *drp-1* RNAi | 113(27) | 24 | 32 | 0.0253^a^ |
|  | *wah-1(bon89)*- C RNAi | 162(55) | 20 | 30 | <0.0001^a^ |
|  | *wah-1(bon89)*- *lrk-1* RNAi | 152(55) | 24 | 34 | ns^a^  <0.0001^b^ |
|  | *wah-1(bon89)*- *drp-1* RNAi | 153(44) | 24 | 34 | ns^a^  <0.0001^b^ |

^a^*p* value compared to wt (N2)- C RNAi

^b^*p* value compared to *wah-1(bon89)*- C RNAi

| **HT115** | | | | | |
| --- | --- | --- | --- | --- | --- |
| **Exp** | **Genotypes and treatments** | **Dead (Censored)** | **Median** | **Max** | ***p* value** |
| 31 | wt (N2)- C RNAi | 73(33) | 25 | 34 | - |
|  | wt (N2)- *pink-1* RNAi | 67(33) | 23 | 32 | ns^a^ |
|  | *wah-1(bon89)-* C RNAi | 84(36) | 20 | 30 | <0.0001^a^ |
|  | *wah-1(bon89)- pink-1* RNAi | 86(64) | 23 | 36 | 0.0024^a^  <0.0001^b^ |
| 32 | wt (N2)- C RNAi | 115(20) | 24 | 41 | - |
|  | wt (N2)- *pink-1* RNAi | 85(36) | 24 | 35 | 0.0004^a^ |
|  | *wah-1(bon89)-* C RNAi | 137(9) | 20 | 35 | 0.0001^a^ |
|  | *wah-1(bon89)-* *pink-1* RNAi | 89(42) | 22 | 35 | 0.0221^a^  ns^b^ |
| 33 | wt (N2)- C RNAi | 118(17) | 24 | 32 | - |
|  | wt (N2)- *pink-1* RNAi | 101(22) | 24 | 32 | ns^a^ |
|  | *wah-1(bon89)-* C RNAi | 127(38) | 20 | 32 | <0.0001^a^ |
|  | *wah-1(bon89)-* *pink-1* RNAi | 94(77) | 22 | 32 | 0.0024^a^  <0.0001^b^ |

^a^*p* value compared to wt (N2)- C RNAi

^b^*p* value compared to *wah-1(bon89)* – C RNAi

| **HT115** | | | | | |
| --- | --- | --- | --- | --- | --- |
| **Exp** | **Genotypes and treatments** | **Dead (Censored)** | **Median** | **Max** | ***p* value** |
| 34 | wt (N2)- C RNAi | 93(22) | 24 | 37 | - |
|  | wt (N2)- *lrk-1* RNAi | 80(25) | 24 | 35 | ns^a^ |
|  | *drp-1(tm1108)*- C RNAi | 98(25) | 22 | 33 | <0.0001^a^ |
|  | *drp-1(tm1108)*- *lrk-1* RNAi | 93(34) | 20 | 33 | <0.0001^a^  ns^b^ |
|  | *wah-1(bon89)*- C RNAi | 87(46) | 19 | 30 | <0.0001^a^ |
|  | *wah-1(bon89)*- *lrk-1* RNAi | 68(54) | 23 | 35 | 0.0234^a^  <0.0001^c^ |
|  | *wah-1(bon89);drp-1(tm1108)*- C RNAi | 119(57) | 25 | 35 | ns^a^  <0.0001^c^ |
|  | *wah-1(bon89);drp-1(tm1108)*- *lrk-1* RNAi | 126(55) | 25 | 37 | ns^a^  <0.0001^c^  ns^d^ |
| 35 | wt (N2)- C RNAi | 74(20) | 24 | 37 | - |
|  | wt (N2)- *lrk-1* RNAi | 73(22) | 24 | 35 | ns^a^ |
|  | *drp-1(tm1108)*- C RNAi | 86(44) | 21 | 32 | <0.0001^a^ |
|  | *drp-1(tm1108)-* *lrk-1* RNAi | 95(38) | 24 | 32 | 0.0003^a^  ns^b^ |
|  | *wah-1(bon89)*- C RNAi | 88(50) | 19 | 30 | <0.0001^a^ |
|  | *wah-1(bon89)*- *lrk-1* RNAi | 91(53) | 24 | 35 | ns^a^  <0.0001^c^ |
|  | *wah-1(bon89);drp-1(tm1108)*- C RNAi | 100(47) | 24 | 37 | ns^a^  <0.0001^c^ |
|  | *wah-1(bon89);drp-1(tm1108)*- *lrk-1* RNAi | 127(39) | 24 | 37 | ns^a^  <0.0001^c^  ns^d^ |
| 36 | wt (N2)- C RNAi | 90(27) | 24 | 36 | - |
|  | wt (N2)- *lrk-1* RNAi | 65(26) | 24 | 36 | ns^a^ |
|  | *drp-1(tm1108)*- C RNAi | 91(40) | 22 | 34 | <0.0001^a^ |
|  | *drp-1(tm1108)*- *lrk-1* RNAi | 83(33) | 22 | 34 | <0.0001^a^  ns^b^ |
|  | *wah-1(bon89)*- C RNAi | 68(55) | 19 | 28 | <0.0001^a^ |
|  | *wah-1(bon89)*- *lrk-1* RNAi | 73(73) | 24 | 36 | ns^a^  <0.0001^c^ |
|  | *wah-1(bon89);drp-1(tm1108)*- C RNAi | 115(58) | 24 | 36 | ns^a^  <0.0001^c^ |
|  | *wah-1(bon89);drp-1(tm1108)*- *lrk-1* RNAi | 149(39) | 24 | 38 | ns^a^  <0.0001^c^  ns^d^ |

^a^*p* value compared to wt (N2)- C RNAi

^b^*p* value compared to *drp-1(tm1108)*- C RNAi

^c^*p* value compared to *wah-1(bon89)*- C RNAi

^d^*p* value compared to *wah-1(bon89);drp-1(tm1108)-* C RNAi

| **OP50(xu363)** | | | | | |
| --- | --- | --- | --- | --- | --- |
| **Exp** | **Genotypes and treatments** | **Dead (Censored)** | **Median** | **Max** | ***p* value** |
| 37 | wt (N2)- C RNAi | 97(24) | 24 | 34 | - |
|  | wt (N2)- *wah-1* RNAi | 93(34) | 26 | 38 | 0.0003^a^ |
|  | *DRP-1 O/E-* C RNAi | 76(55) | 23 | 33 | ns^a^ |
|  | *DRP-1 O/E-* *wah-1* RNAi | 94(24) | 21 | 31 | 0.0005^a^  0.0142^b^ |
| 38 | wt (N2)- C RNAi | 104(24) | 24 | 34 | - |
|  | wt (N2)- *wah-1* RNAi | 67(35) | 26 | 36 | 0.0052^a^ |
|  | *DRP-1 O/E-* C RNAi | 78(48) | 23 | 35 | ns^a^ |
|  | *DRP-1 O/E-* *wah-1* RNAi | 78(44) | 21 | 31 | <0.0001^a^  0.0120^b^ |
| 39 | wt (N2)- C RNAi | 113(10) | 24 | 34 | - |
|  | wt (N2)- *wah-1* RNAi | 91(38) | 28 | 36 | 0.0002^a^ |
|  | *DRP-1 O/E-* C RNAi | 74(48) | 26 | 34 | ns^a^ |
|  | *DRP-1 O/E-* *wah-1* RNAi | 82(46) | 22 | 32 | 0.0002^a^ 0.0020^b^ |

^a^*p* value compared to wt (N2)- C RNAi

^b^*p* value compared to *DRP-1 O/E-* C RNAi

| **HT115** | | | | | |
| --- | --- | --- | --- | --- | --- |
| **Exp** | **Genotypes and treatments** | **Dead (Censored)** | **Median** | **Max** | ***p value*** |
| 40 | wt (N2)- C RNAi | 79(13) | 24 | 30 | - |
|  | wt (N2)- *drp-1* RNAi | 71(24) | 24 | 28 | ns^a^ |
|  | *lrk-1(bon150)*- C RNAi | 60(3) | 24 | 34 | ns^a^ |
|  | *lrk-1(bon150)*- *drp-1* RNAi | 75(10) | 24 | 31 | ns^a^  ns^b^ |
|  | *lrk-1(bon151)*- C RNAi | 126(15) | 24 | 30 | ns^a^ |
|  | *lrk-1(bon151)*- *drp-1* RNAi | 117(9) | 24 | 30 | 0.0027^a^  <0.0001^c^ |
|  | *lrk-1(bon150);wah-1(bon89)*- C RNAi | 33(50) | 24 | 38 | ns^a^  ns^b^ |
|  | *lrk-1(bon150);wah-1(bon89)-*  *drp-1* RNAi | 82(34) | 24 | 38 | 0.005^a^  ns^b^ |
|  | *lrk-1(bon151);wah-1(bon89)*- C RNAi | 22(32) | 26 | 36 | 0.0087^a^  0.0003^c^ |
|  | *lrk-1(bon151);wah-1(bon89)*- *drp-1* RNAi | 34(7) | 24 | 36 | 0.0002^a^  0.0003^c^ |
| 41 | wt (N2)- C RNAi | 103(11) | 24 | 33 | - |
|  | wt (N2)- *drp-1* RNAi | 111(7) | 24 | 33 | ns^a^ |
|  | *lrk-1(bon150)*- C RNAi | 102(18) | 24 | 33 | ns^a^ |
|  | *lrk-1(bon150)*- *drp-1* RNAi | 113(22) | 24 | 28 | ns^a^  0.0017^b^ |
|  | *lrk-1(bon151)*- C RNAi | 121(20) | 24 | 31 | ns^a^ |
|  | *lrk-1(bon151)*- *drp-1* RNAi | 129(10) | 24 | 37 | 0.0111^a^  0.0002^b^ |
|  | *lrk-1(bon150);wah-1(bon89)*- C RNAi | 50(111) | 24 | 31 | ns^a^  ns^b^ |
|  | *lrk-1(bon150);wah-1(bon89)*- *drp-1* RNAi | 80(74) | 24 | 33 | 0.0048^a^  ns^b^ |
|  | *lrk-1(bon151);wah-1(bon89)*- C RNAi | 32(58) | 26 | 33 | 0.074^a^  0.015^c^ |
|  | *lrk-1(bon151);wah-1(bon89)*- *drp-1* RNAi | 49(59) | 24 | 33 | ns^a^  0.0255^c^ |
| 42 | wt (N2)- C RNAi | 78(29) | 24 | 35 | - |
|  | wt (N2)- *drp-1* RNAi | 90(19) | 22 | 31 | 0.0029^a^ |
|  | *lrk-1(bon150)*- C RNAi | 88(24) | 24 | 33 | ns^a^ |
|  | *lrk-1(bon150)-* *drp-1* RNAi | 91(13) | 24 | 35 | ns^a^ |
|  | *lrk-1(bon151)*- C RNAi | 92(23) | 24 | 33 | 0.0463^a^ |
|  | *lrk-1(bon151)*- *drp-1* RNAi | 96(20) | 24 | 35 | ns^a^  0.0048^c^ |
|  | *lrk-1(bon150);wah-1(bon89)*- C RNAi | 68(82) | 24 | 32 | ns^a^ |
|  | *lrk-1(bon150);wah-1(bon89)*- *drp-1* RNAi | 84(66) | 26 | 33 | 0.0009^a^  <0.0001^b^ |
|  | *lrk-1(bon151);wah-1(bon89)*- C RNAi | 46(98) | 24 | 32 | ns^a^  ns^c^ |
|  | *lrk-1(bon151);wah-1(bon89)*- *drp-1* RNAi | 62(77) | 26 | 34 | ns^a^  0.0104^c^ |
| 43 | wt (N2)- C RNAi | 73(36) | 25 | 35 | - |
|  | wt (N2)- *drp-1* RNAi | 99(24) | 23 | 33 | 0.0034^a^ |
|  | *lrk-1(bon150)*- C RNAi | 66(40) | 25 | 35 | ns^a^ |
|  | *lrk-1(bon150)*- *drp-1* RNAi | 72(41) | 25 | 35 | ns^a^  ns^b^ |
|  | *lrk-1(bon151)*- C RNAi | 67(49) | 25 | 35 | ns^a^ |
|  | *lrk-1(bon151)*- *drp-1* RNAi | 75(42) | 25 | 35 | ns^a^  ns^c^ |
|  | *lrk-1(bon150);wah-1(bon89)*- C RNAi | 66(84) | 25 | 35 | ns^a^  ns^b^ |
|  | *lrk-1(bon150);wah-1(bon89)*- *drp-1* RNAi | 96(54) | 25 | 35 | ns^a^  ns^b^ |
|  | *lrk-1(bon151);wah-1(bon89)*- C RNAi | 59(80) | 25 | 35 | ns^a^  ns^c^ |
|  | *lrk-1(bon151);wah-1(bon89)*- *drp-1* RNAi | 69(79) | 25 | 35 | ns^a^  ns^c^ |

^a^*p* value compared to wt (N2)- C RNAi

^b^*p* value compared to *lrk-1(bon150)*- C RNAi

^c^*p* value compared to *lrk-1(bon151)*- C RNAi
