# Supplementary material for: Dietary lipid content modifies wah-1/AIFM1-associated phenotypes via LRK-1 and DRP-1 expression in C. elegans
Source: Nat Commun. 2025 Dec 1;16:10817. doi: 10.1038/s41467-025-66900-8 (PMC12669733; doi:10.1038/s41467-025-66900-8)
Supplement: Supplementary file 10 — Supplementary data 8 [file 41467_2025_66900_MOESM10_ESM.zip › AIF-WAH-1_Supplementary data 8.docx]

**Supplementary data 8.**

Genotypes, number of animals (dead and censored subjects), median survival (days) and max (days) lifespan are reported in the table for each individual experiment (Exp). Log-rank (Mantel-Cox) test was used to calculate *p* values (relative to C RNAi).

| **HT115** | | | | | |
| --- | --- | --- | --- | --- | --- |
| **Exp** | **Genotypes and treatments** | **Dead (Censored)** | **Median** | **Max** | ***p* value** |
| 1 | *wah-1(bon89)-* C RNAi | 51(62) | 20 | 31 | - |
|  | *wah-1(bon89)-* *alh-13* RNAi | 106(19) | 25 | 40 | <0.0001^a^ |
|  | *wah-1(bon89)-* *atad-3* RNAi | 60(25) | 29 | 39 | <0.0001^a^ |
|  | *wah-1(bon89)- chch-3* RNAi | 84(33) | 20 | 31 | ns^a^ |
|  | *wah-1(bon89)-* *clk-1* RNAi | 42(58) | 20 | 29 | ns^a^ |
|  | *wah-1(bon89)-* *coa-7* RNAi | 42(59) | 23 | 33 | ns^a^ |
|  | *wah-1(bon89)-* *stl-1* RNAi | 60(50) | 20 | 37 | 0.0325^a^ |
|  | *wah-1(bon89)- tin-44* RNAi | 89(24) | 27 | 37 | <0.0001^a^ |
|  | *wah-1(bon89)-* *tomm-70* RNAi | 63(51) | 20 | 31 | ns^a^ |
|  | *wah-1(bon89)- tsfm-1* RNAi | 56(60) | 23 | 35 | 0.0170^a^ |
| 2 | *wah-1(bon89)*- C RNAi | 53(29) | 19 | 29 | - |
|  | *wah-1(bon89)*- *aak-2* RNAi | 50(28) | 21 | 34 | 0.0437^a^ |
|  | *wah-1(bon89)*- *aco-2* RNAi | 60(20) | 25 | 39 | <0.0001^a^ |
|  | *wah-1(bon89)*- *dtmk-1* RNAi | 50(51) | 23 | 32 | 0.0295^a^ |
|  | *wah-1(bon89)*- *gba-1* RNAi | 58(28) | 23 | 32 | 0.0042^a^ |
|  | *wah-1(bon89)*- *mppa-1* RNAi | 77(19) | 25 | 36 | <0.0001^a^ |
|  | *wah-1(bon89)*- *pcca-1* RNAi | 50(38) | 21 | 29 | 0.0291^a^ |
|  | *wah-1(bon89)*- *rad-8* RNAi | 49(32) | 23 | 32 | 0.0001^a^ |

^a^*p* value compared to *wah-1(bon89)-* C RNAi

| **HT115** | | | | | |
| --- | --- | --- | --- | --- | --- |
| **Exp** | **Genotypes and treatments** | **Dead (Censored)** | **Median** | **Max** | ***p* value** |
| 3 | wt(N2)- C RNAi | 63(21) | 24 | 35 | - |
|  | wt(N2)- *aco-2* RNAi | 53(29) | 24 | 35 | ns^a^ |
|  | wt(N2)- *alh-13* RNAi | 71(20) | 28 | 39 | <0.0001^a^ |
|  | wt(N2)- *atad-3* RNAi | 58(25) | 28 | 41 | <0.0001^a^ |
|  | wt(N2)- *dtmk-1* RNAi | 73(12) | 26 | 39 | 0.0111^a^ |
|  | wt(N2)- *gba-1* RNAi | 61(25) | 26 | 35 | 0.0319^a^ |
|  | wt(N2)- *mppa-1* RNAi | 70(14) | 24 | 33 | ns^a^ |
|  | wt(N2)- *rad-8* RNAi | 58(23) | 26 | 37 | 0.0036^a^ |
|  | wt(N2)- *tin-44* RNAi | 63(23) | 26 | 39 | 0.0154^a^ |

^a^*p* value compared to wt (N2)- C RNAi

| **HT115** | | | | | |
| --- | --- | --- | --- | --- | --- |
| **Exp** | **Genotypes and treatments** | **Dead (Censored)** | **Median** | **Max** | ***p* value** |
| 4^b^ | *wah-1(bon89)-* C RNAi | reported in Supplementary table ST1 | | | |
|  | *wah-1(bon89)-* *drp-1* RNAi | reported in Supplementary table ST1 | | | |
|  | *wah-1(bon89)-* *lrk-1* RNAi | reported in Supplementary table ST1 | | | |
|  | wt(N2)*-* C RNAi | reported in Supplementary table ST1 | | | |
|  | wt(N2)*-* *drp-1* RNAi | reported in Supplementary table ST1 | | | |
|  | wt(N2)*-* *lrk-1* RNAi | reported in Supplementary table ST1 | | | |

^b^In ST1, this experiment is number 28.
